# Oral pre-treatment with thiocyanate (SCN^−^) protects against myocardial ischaemia–reperfusion injury in rats

**DOI:** 10.1038/s41598-021-92142-x

**Published:** 2021-06-16

**Authors:** Luke Hall, Chaouri Guo, Sarah Tandy, Kathryn Broadhouse, Anthony C. Dona, Ernst Malle, Emil D. Bartels, Christina Christoffersen, Stuart M. Grieve, Gemma Figtree, Clare L. Hawkins, Michael J. Davies

**Affiliations:** 1 The Heart Research Institute, Newtown, NSW 2042 Australia; 2 School of Medicine, University of Sydney, Sydney, NSW 2006 Australia; 3 Department of Biomedical Sciences, Panum Institute, University of Copenhagen, Blegdamsvej 3, 2200 Copenhagen, Denmark; 4 Northern Medical School, Kolling Institute of Medical Research, University of Sydney, Sydney, NSW 2065 Australia; 5 Division of Molecular Biology and Biochemistry, Gottfried Schatz Research Center, Medical University of Graz, Graz, Austria; 6 Department of Clinical Biochemistry, Copenhagen University Hospital Rigshospitalet, Copenhagen, Denmark

**Keywords:** Atherosclerosis, Coronary artery disease and stable angina, Chemical modification

## Abstract

Despite improvements in revascularization after a myocardial infarction, coronary disease remains a major contributor to global mortality. Neutrophil infiltration and activation contributes to tissue damage, via the release of myeloperoxidase (MPO) and formation of the damaging oxidant hypochlorous acid. We hypothesized that elevation of thiocyanate ions (SCN^−^), a competitive MPO substrate, would modulate tissue damage. Oral dosing of rats with SCN^−^, before acute ischemia–reperfusion injury (30 min occlusion, 24 h or 4 week recovery), significantly reduced the infarct size as a percentage of the total reperfused area (54% versus 74%), and increased the salvageable area (46% versus 26%) as determined by MRI imaging. No difference was observed in fractional shortening, but supplementation resulted in both left-ventricle end diastolic and left-ventricle end systolic areas returning to control levels, as determined by echocardiography. Supplementation also decreased antibody recognition of HOCl-damaged myocardial proteins. SCN^−^ supplementation did not modulate serum markers of damage/inflammation (ANP, BNP, galectin-3, CRP), but returned metabolomic abnormalities (reductions in histidine, creatine and leucine by 0.83-, 0.84- and 0.89-fold, respectively), determined by NMR, to control levels. These data indicate that elevated levels of the MPO substrate SCN^−^, which can be readily modulated by dietary means, can protect against acute ischemia–reperfusion injury.

## Introduction

Cardiovascular disease is a leading cause of death worldwide^1^. Recent research has targeted both primary and secondary means of prevention in high risk populations, and also driven advances in medical interventions for cardiovascular diseases (e.g.^2,3^). A number of animal models that simulate human cardiac disease have been developed, including myocardial infarction (MI) and ischemia–reperfusion (IR) models that induce heart failure (HF)^4^. Rats are a commonly employed experimental system^5^, with simulation of MI achieved by transient ligation of the left anterior descending (LAD) coronary artery^6^. The LAD ligation may be tightened temporarily and removed after a given period of time (e.g. 30 min) to simulate an acute event, or tied off indefinitely to simulate a chronic (permanent) MI. The duration of reperfusion following a non-permanent ligation can also be altered. A short reperfusion period (24 h) is typically used in order to determine infarct size and salvageable area (or area at risk, AAR), whereas prolonged periods (4 weeks) can be used to evaluate changes in cardiac function and re-modelling. The degree of re-modelling (apoptosis, necrosis and ventricular dysfunction) depends on both the length of the ischemia and reperfusion periods^4^.

Acute obstruction of the coronary artery results in the affected region of myocardium experiencing acute ischemia, and in so doing determines the salvageable area of the MI if the coronary occlusion is temporary. The deprivation of both O_2_ and nutrients results in a cascade of rapid metabolic and biochemical changes, including the cessation of oxidative phosphorylation, which results in depolarization of the mitochondrial membrane, depletion of ATP, and inhibition of myocardial contractility. The absence of O_2_ as a terminal electron acceptor also switches cellular metabolism to anaerobic glycolysis, which results in intracellular lactate accumulation, proton accumulation and a reduction in pH^7,8^. This activates the Na^+^–H^+^ ion exchanger, extruding protons from the cell in exchange for Na^+^. ATP depletion during ischemia also deactivates the 3Na^+^–2K^+^ ATPase, and therefore exacerbating the intracellular Na^+^ overload. This activates the reverse function of the 2Na^+^–Ca^2+^ ion exchanger, as the cell tries to extrude Na^+^ but results in intracellular Ca^2+^ overload^9^. Thus, ischemic periods of beyond a few minutes results in cardiomyocyte death which starts in the subendocardium and spreads as a ‘wavefront’ transmurally toward the epicardium^10^.

Prompt myocardial reperfusion is crucial for the salvage of viable myocardium, to limit MI size, preserve left-ventricle systolic function, and prevent the onset of heart failure. However, while reperfusion represents the absolute treatment for acutely ischemic myocardium, it can also induce cardiomyocyte death^11^ via a number of pathways including reperfusion-induced arrhythmias^12^, myocardial stunning^13^, microvascular obstruction^14^, and lethal myocardial reperfusion injury^15^**.**

Neutrophil infiltration and subsequent activation is a primary response in IR injury, with these cells implicated in mediating lethal injury (reviewed^16,17^). Ischemia without reperfusion results in slow neutrophil infiltration into the salvageable area over a 12–24 h period. With reperfusion, neutrophil infiltration and accumulation is accelerated and increased^16,17^. The activation of neutrophils results in the release of reactive oxidants, proteases (e.g. elastase, collagenase, gelatinase and cathepsins which target extracellular matrix proteins), enzymes (such as the abundant heme enzyme, myeloperoxidase; MPO), lipids and cytokines, which alter the expression of other pro-inflammatory mediators and adhesion molecules^16–18^. Thus, the recruitment of neutrophils (which contain up to 5% MPO by total protein mass) at the onset of reperfusion initiates a cascade of cell–cell interactions, trans-endothelial migration, and reaction with myocytes^19^.

The over-production of oxidants by activated neutrophils, particularly hypochlorous acid (HOCl), which is generated by MPO from H_2_O_2_ and Cl^−^, contributes to the damage sustained by the myocardium following reperfusion (reviewed^7,8,20^). While Cl^−^ is the most abundant substrate for MPO, other halide (bromide, Br^−^; iodide, I^−^) and pseudohalide (thiocyanate, SCN^−^) ions can also be oxidized yielding hypobromous acid, hypoiodous acid and hypothiocyanous acid (HOSCN), respectively (reviewed^21^). At physiological pH and halide ion concentrations (~ 100 mM Cl^−^, 50 µM Br^−^, 50 µM SCN^−^), MPO predominantly generates HOCl and HOSCN (~ 50–55% and ~ 40% of the H_2_O_2_ consumed, respectively), due to the higher specificity constant of MPO for SCN^−^ compared to Cl^−^ (730:1)^22^. However, the overall yield of each oxidant will vary between individuals, as human plasma SCN^−^ concentrations are dependent on a number of factors including smoking status and diet^21,23,24^. This is significant because of the marked difference in chemical reactivity and biological effects of HOCl compared to HOSCN^21,24^. Previous studies in rodents have shown that oral dosing with SCN^−^ in drinking water for 7 days or more, results in a significant elevation (> twofold) of plasma/serum SCN^−^ concentrations^25,26^, and that this remains elevated, at a plateau value, for long periods with continued dosing^26^. After cessation of dosing, SCN^−^ concentrations decrease slowly due to the long plasma half-life of this anion (cf. reported t_1/2_ values of between 50 h (after cyanide exposure)^27^ and 14 days^28^.

HOCl is a potent oxidant that reacts rapidly with most biological substrates, and it is strongly linked with the induction of tissue damage in chronic inflammatory pathologies^21,29–32^, including the heart after MI^33^. In contrast, HOSCN is a weaker oxidant that reacts less rapidly, and in a (mostly) reversible manner, with thiol and seleno compounds^34–36^, and therefore can be detoxified by some cellular antioxidant enzymes^23,37^. As a consequence, decreasing HOCl formation, by increasing the availability of SCN^−^ (with consequent HOSCN generation) has been proposed as a mechanism to alleviate damage induced by MPO at sites of inflammation^23,26,38^. A number of studies have reported positive effects of increased SCN^−^ levels in chemical, cellular and in vivo chronic inflammation systems (e.g.^23,26,38–43^). Whether elevated SCN^−^ concentrations can modulate damage generated by acute IR injury has not been examined in detail, though HOSCN has been shown to be less toxic to cardiac myocytes than HOCl in vitro^44^. In addition, high SCN^−^ levels have been reported to decrease all-cause mortality in a retrospective analysis of a human cohort that survived an initial MI^39^. The hypothesis for the study reported here was therefore that high levels of SCN^−^ might afford protection against IR-induced myocardial damage.

## Results

### Thiocyanate (SCN^−^) supplementation

**Figure 1:**
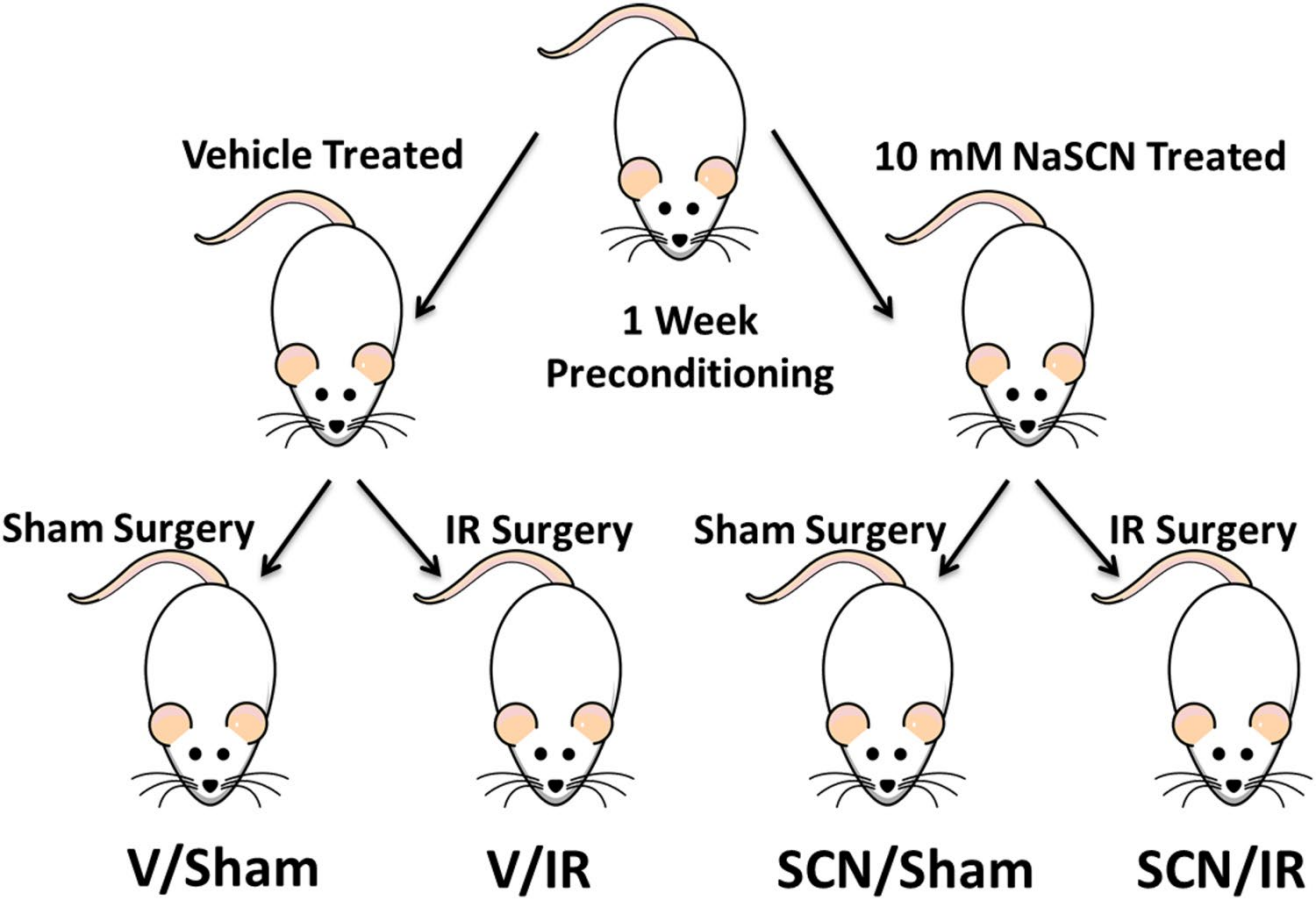
Rat IR experimental model. Sprague–Dawley rats were preconditioned for one week with either regular water or 10 mM NaSCN before the start of the experiments. They then underwent either sham or IR surgery to give 4 groups; vehicle (V)-treated shams (V/Sham), V-treated IR (V/IR), SCN^–^ treated shams (SCN/Sham), and SCN^–^ treated IR (SCN/IR). After sham or IR surgery the animals were allowed to recover for 24 h or 4 weeks before sacrifice and analysis.

**Figure 2:**
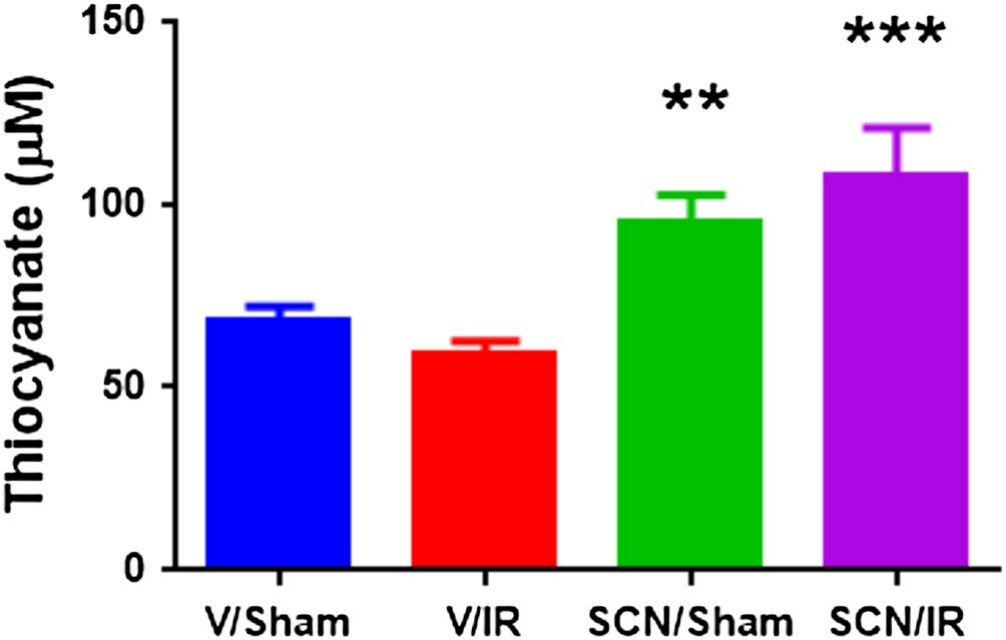
Serum SCN^−^ levels at 24 h post-surgery. A significant elevation in serum SCN^−^ levels was observed for both the SCN^−^-treated groups as determined by one-way ANOVA with a post-hoc Newman-Keuls Multiple Comparison Test, where ** = p < 0.01 and *** = p < 0.001, relative to the corresponding controls.

Figure 1 gives an overview of the experimental design and the supplementation protocol. To confirm the efficacy of supplementation, rat serum SCN^−^ levels were measured by ion-exchange chromatography. The mean SCN^−^ concentrations for each treatment group, at 24 h post-surgery, are presented in Fig. 2. These data indicate that serum SCN^−^ levels were significantly elevated in the treatment groups consistent with previous studies^25,26^. As this anion has a long plasma half-life (see above) a significant elevation would be expected for a considerable period after the tissue injury.

### Magnetic resonance imaging of tissue damage

**Figure 3:**
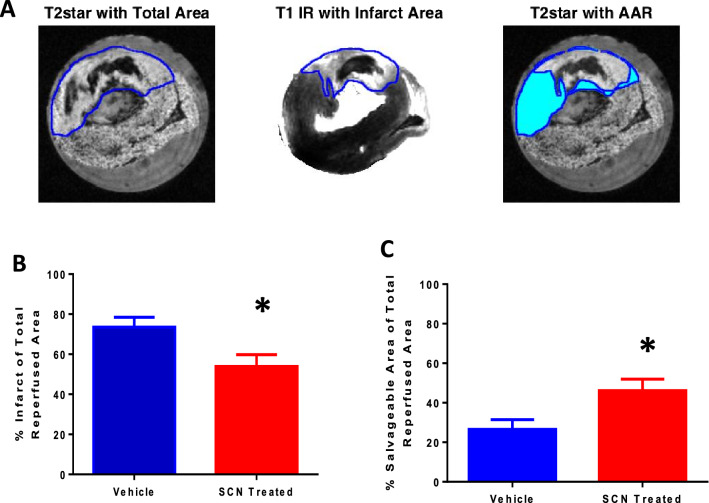
**(A)** Representative images of: (left) the total reperfusion area as determined by T2star MRI image analysis; (middle) infarct area determined by the T1 MRI image analysis; and (right) overlay of the T2star and T1 maps highlighting the salvageable area (AAR) in blue, at 24 h post IR surgery. **(B**) Area of the infarct as a percentage of total reperfusion area for the vehicle IR and SCN^–^ treated IR groups. **(C)** Salvageable area as a percentage of total reperfusion area for the vehicle IR and SCN^–^ treated IR groups. * indicates a statistically-significant difference at the p < 0.05 level between the vehicle and SCN^–^ treated rats subjected to IR injury, using a one-tailed Student’s t-test.

MRI analysis was performed to determine the infarct and salvageable areas after IR injury. Figure 3A shows a representative sample of the MRI output, exhibiting total reperfusion area, infarct area, and salvageable area. Both the infarct and salvageable areas are expressed as percentages of the total reperfusion area to compensate for variation between individual animals and surgeries. Figure 3B indicates that there was a significant reduction in the infarct size as a percentage of the total reperfused area in the SCN^−^–treated rats compared to vehicle controls, and Fig. 3C depicts the concomitant significant increase in salvageable area. The mean control infarct size constituted 74% (and 26% salvageable area), while the mean infarct size in the SCN^−^ -supplemented animals constituted 54% (and 46% salvageable area) of the total reperfusion area, indicating a 20% protection against permanent myocardial damage.

### Echocardiographic analysis of tissue function

**Figure 4:**
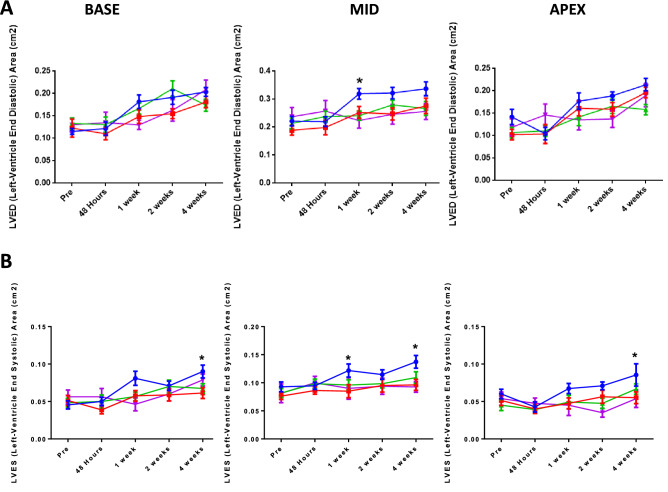
**(A)** Left-ventricle end diastolic area over (left to right) the base, mid and apex sections from pre-surgery to 4 weeks post-surgery for the 4 experimental groups. Red lines and symbols: vehicle/sham group; blue lines and symbols: vehicle/IR group; pink lines and symbols: SCN^−^/sham group; green line and symbols: SCN^−^/IR group. Statistical analysis using two-way ANOVA and Dunnett’s Multiple Comparison Test, showed a significant difference in the mid-section for the vehicle /IR group compared to the other groups at 1 week, where * indicates p < 0.05. **(B)** Left-ventricle end systolic area over (left to right) the base, mid and apex sections from pre-surgery to 4 weeks post-surgery. Colour coding as panel (**A)**. Statistical analysis using two-way ANOVA and Dunnett’s Multiple Comparison Test, showed statistically-significant differences in the vehicle/IR group compared to the other groups at 4 weeks in the base section, 2 and 4 weeks in the mid-section, and at 4 weeks in the apex section, with * indicating p < 0.05.

Echocardiography provides a non-invasive means of assessing cardiac output, with this used to assess multiple parameters over a 4 week period after IR at three different sites; the base, mid and apex. Supplementary Fig. 1 depicts the change in fractional shortening over time for each section of the heart, and for each of the 4 groups. No significant differences were observed with either recovery time or treatment. Figure 4A depicts the change in left-ventricle end diastolic (LVED) area. In both the mid and apex sections from 1 week after surgery, the vehicle/IR group shows a trend towards a greater LVED area when compared to the other 3 groups, with this being significant at 1 week post-surgery, in the mid-section. The SCN^–^-treated IR group was not significantly different to the control vehicle/sham indicating a protection against damage.

Figure 4B depicts the change in left-ventricle end systolic (LVES) area. Across all sections from 1 week post-surgery, the vehicle/IR group showed a trend towards a greater LVES area than the 3 other groups, with the SCN^–^-treated IR group closely paralleling the vehicle/sham group. Significant differences were observed in the base section at 4 weeks, the mid-section at 1 and 4 weeks, and the apex at 4 weeks when compared to the vehicle/IR group. As increased LVED and LVES area is functionally detrimental, and associated with worse prognostic outcomes including HF^45,46^, the observed increase in the LVED area detected for the mid-section of the heart for the vehicle/IR group, and the increase in LVES area for the same group at all regions of the heart is consistent with functional damage engendered by the IR injury. Conversely, the absence of any significant differences compared to the controls for the SCN/IR group is consistent with the elevated SCN^−^ affording protection against ventricle chamber dilatation, and possible HF following MI/IR injury in this model.

### Histologic evaluation of tissue damage

**Figure 5:**
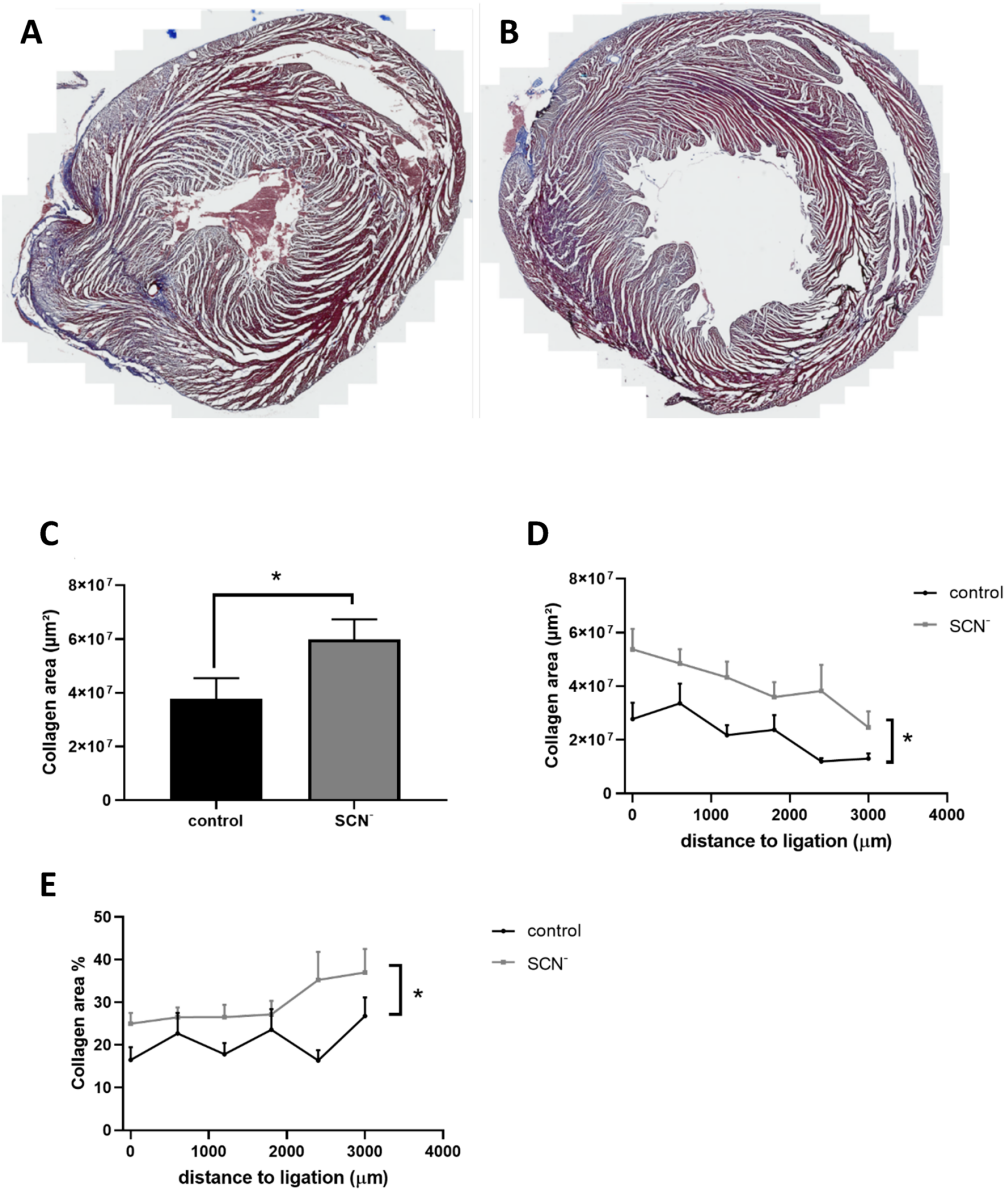
Supplementation with SCN^−^ increases content of collagen. Panels (**A**) and (**B**) show representative Trichrome-stained images taken at the site of ligation at 24 h post surgery from: (**A**) a vehicle/IR rat, and (**B**) a SCN^–^supplemented/IR rat, with the blue staining depicting collagen. (**C**) shows the quantification of collagen areas from 6 sections taken between the ligation site and apex in each heart from the control (black bar, n = 7 rats) and SCN^–^supplemented (grey bar, n = 6 rats) groups with * showing a significant (p < 0.05) difference between groups using a one-tailed t-test. (**D**) and (**E**) Quantification of collagen area was performed on individual sections taken from the site of ligation to the apex for control (black line) and SCN^−^ (grey line) groups with data in (E) expressed as percentage of the total stained area in the section. Data represent mean ± SEM of n ≥ 6 rats with * showing a significant difference between the vehicle/IR animals and the SCN^−^ -supplemented/IR group, using 2-way ANOVA with a Sidak post-hoc test.

As enhanced collagen levels are associated with myocardial remodelling following infarction, possible changes in collagen content arising from the presence of SCN^−^ was examined in the hearts subjected to IR (Fig. 5). No analyses were carried out on the control (no IR) groups due to the absence of an infarct. The data obtained indicate that SCN^−^ supplementation significantly increased the collagen content of the heart, as indicated by an increase in Trichrome staining (Fig. 5A,B). Figure 5C shows the increase in collagen area calculated by combining the data from 6 serial sections cut from the ligation site to the apex from hearts from 7 (control) or 6 (SCN^−^) rats. An increase in collagen on supplementation with SCN^−^ was apparent in all regions of the heart as shown by the collagen area (in µm) of each section (Fig. 5D) and the percentage of collagen compared to the total area (Fig. 5E).

**Figure 6:**
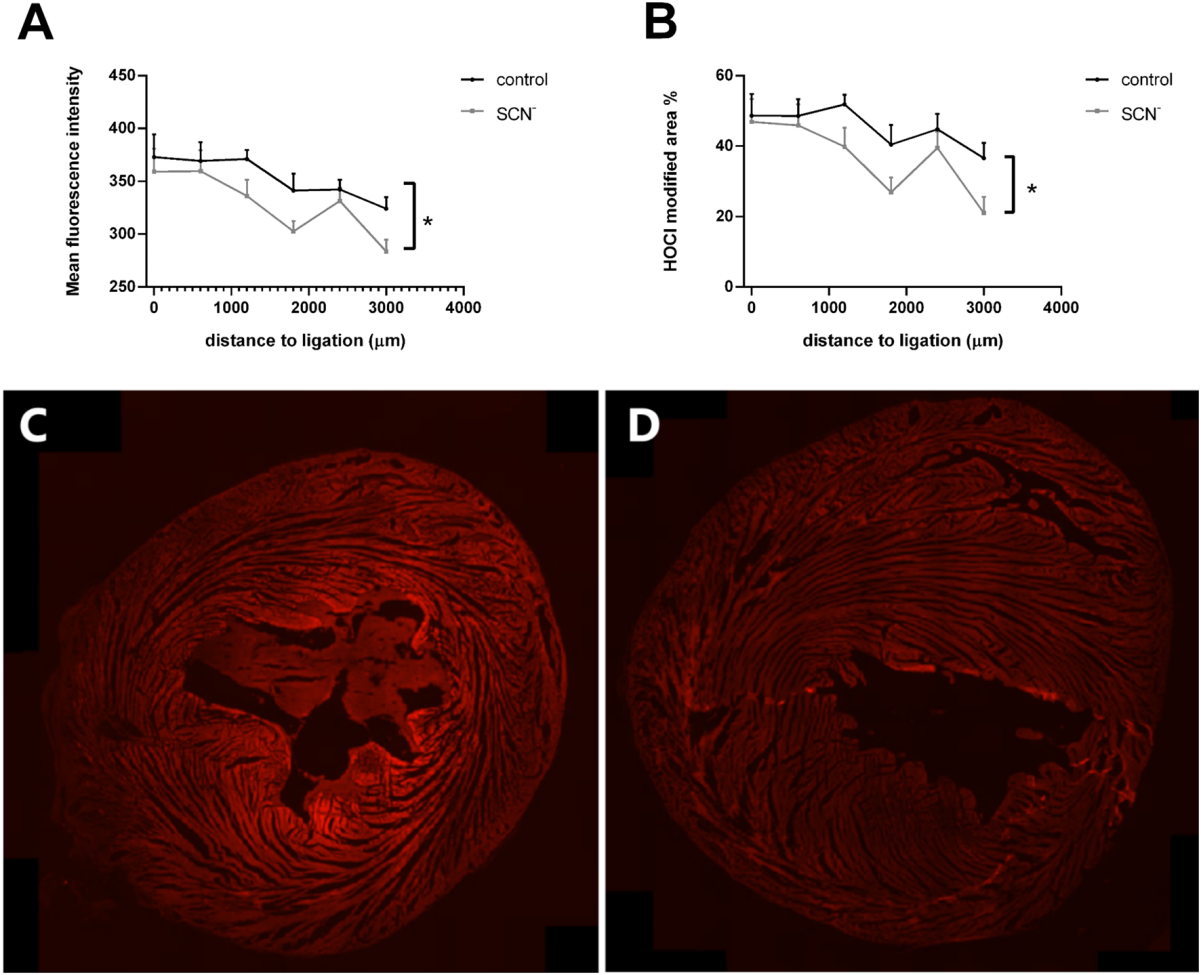
Effect of SCN^−^ supplementation on the extent of formation of HOCl-modified proteins. The graphs show the differences in: (**A**) mean fluorescence intensity from 2D10G9 antibody epitopes, and (**B**) the percentage of fluorescence with an intensity over 350 compared to the background fluorescence (over 150) of the individual section, as a representation of the extent of 2D10G9 epitope recognition for HOCl-modified proteins, as measured on sections taken from the ligation site to the apex for control (black line) and SCN^−^ (grey line) groups. Data represent mean ± SEM of n ≥ 6 rats with * showing a significant difference between vehicle/IR and SCN^–^ supplemented/IR groups using 2-way ANOVA with a Sidak post-hoc test. Panels C and D show representative fluorescent images of the 2D10G9 epitopes taken 1800 µm from the site of ligation from: (C) a vehicle/IR rat, and (D) a SCN^–^ supplemented/IR rat, with the images recorded and displayed with the same instrument settings.

The extent of HOCl-mediated damage to myocardial proteins was examined using the murine monoclonal antibody (mAb) 2D10G9, which recognizes proteins modified by this oxidant^47,48^. Due to the presence of weak background fluorescence, a threshold was set to determine sites of increased antibody recognition (see Materials and Methods). An increase in fluorescence intensity consistent with 2D10G9 epitope recognition was observed in sections proximal to the site of ligation, consistent with the production of HOCl. This progressively decreased at greater distances from the ligation site (Fig. 6). Supplementation with SCN^−^ had no significant effect on the extent of 2D10G9 recognition at the ligation site, as determined by the fluorescence intensity relative to controls (Supplementary Fig. 2), but the decrease in 2D10G9 epitope recognition diminished more rapidly in sections increasingly distant to the ligation site in the SCN^–^supplemented animals (Fig. 6).

Possible correlations between collagen content and the extent of mAb 2D10G9 recognition was examined (Supplementary Fig. 3). These two variables showed a significant negative correlation for both the vehicle/IR and SCN/IR groups, when considered as total cohort, with r values of -0.5057 and -0.3878 respectively, though there was variation between animals. However, all the vehicle/IR animals and all except one animal in the SCN/IR group, showed a negative correlation (Supplementary Table 1).

### Serum analyses of circulating biomarkers of heart failure and inflammation

Quantification of the levels of atrial natriuretic peptide (ANP) and brain natriuretic peptide (BNP), well established biomarkers of HF^49^, showed no significant differences between the 4 groups at either 24 h or 4 weeks post-surgery (Supplementary Fig. 4A and B). Similarly, there were no significant differences in galectin-3 levels (Supplementary Fig. 4C), which is secreted by macrophages and strongly linked with cardiac fibrosis^50^ or the acute phase protein, C-reactive protein (CRP)^51^ (Supplementary Fig. 4D). However, a significant reduction in CRP levels was detected for the 4 week post-surgery groups compared to the 24 h groups, consistent with the short (~ 19 h) biological half-life of this protein^52^.

### Metabolomic analyses of serum

**Figure 7:**
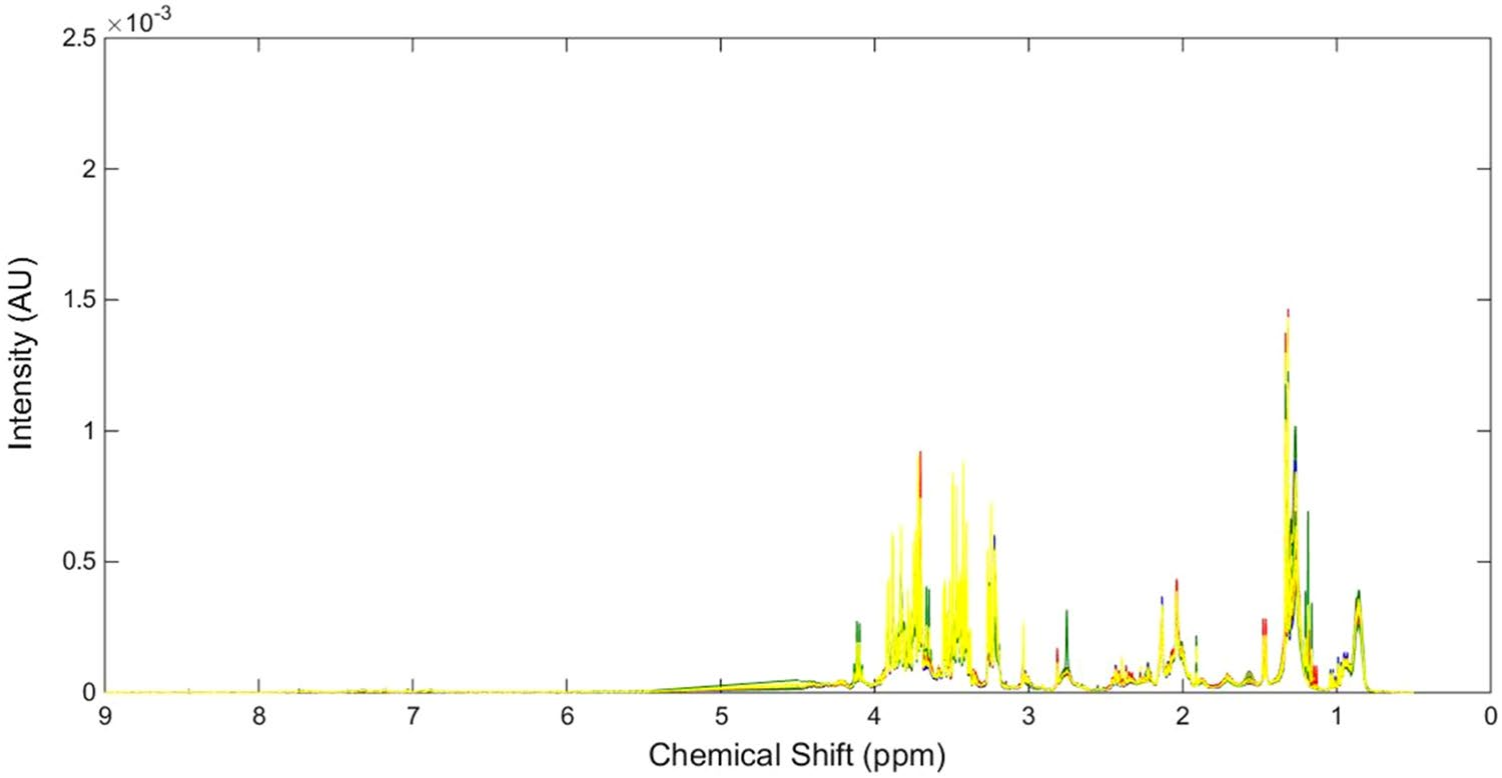
Representative NMR spectra for serum samples obtained from vehicle/sham (blue), vehicle/IR (red), SCN^–^ supplemented/sham (green), and SCN^–^supplemented/IR (yellow) animals at 24 h post-surgery.

NMR metabolomic analysis was performed on serum samples from the four treatment groups at 24 h post IR surgery to assess levels of individual metabolites in the absence and presence of IR and / or SCN^−^ (Fig. 7). Spectral peaks were assigned to particular species using literature data and the Chenomx NMR Suite (see Materials and Methods). A list of the identified metabolites, their assigned chemical shifts (ppm), and average fold changes with time and either IR or sham surgery, and treatment are detailed in Table 1 for the 24 h samples and Table 2 for the 4 week animals. Statistically-significant reductions in several metabolites were detected for the vehicle/IR group relative to vehicle/sham group at 24 h post-surgery. Namely, phenylalanine (7.40 ppm) had a fold change of 0.82, acetate (1.91 ppm) had a fold change of 0.83, and valine (1.03 ppm) had a fold change of 0.90. In contrast, the SCN/IR group only showed a statistically-significant increase relative to the SCN/sham control, at 24 h post-surgery, in regard to glutamine (2.46 ppm) levels, which had a fold change of 1.15. No significant differences in metabolite expression were observed between the SCN/IR and vehicle/IR groups.

**Table 1:** Concentrations of major endogenous metabolites quantified in rat serum by ^1^H-NMR, and their change in expression in vehicle/IR and SCN^−^-supplemented/IR rats at 24 h following surgery.

Metabolite	Chemical Shift (ppm)	Average fold change at 24 h: vehicle/sham versus vehicle/IR	Average fold change at 24 h: SCN/sham versus SCN/IR	Average fold change at 24 h: vehicle/IR versus SCN/IR
Formate	8.52	0.95	1.41	1.02
Cytidine	7.80	0.93	0.97	0.96
Methylhistidine	7.74	0.88	1.08	0.95
Histidine	7.71	0.98	1.07	1.08
Phenylalanine	7.40	***0.82***	1.09	0.94
Tyrosine	7.17	0.89	1.05	0.96
Lactate	4.11	1.01	1.01	1.11
Isoleucine	3.65	0.95	0.99	0.99
Creatinine/creatinine phosphate	3.03	0.94	1.13	0.95
Creatine	3.00	0.95	1.08	0.95
Trimethylamine	2.91	1.02	1.06	1.00
Methylguanidine	2.81	1.08	1.07	0.97
Methionine	2.64	0.95	1.07	0.98
Glutamine	2.46	0.98	***1.15***	1.00
Acetoacetate	2.22	1.18	0.84	1.05
Acetyl Groups (1)	2.13	0.96	1.07	0.98
Acetyl Groups (2)	2.06	1.01	1.02	1.03
Acetate	1.91	***0.83***	0.89	0.95
Leucine	1.72	0.95	1.09	0.97
Lipids (VLDL)	1.57	1.23	0.82	1.04
Alanine	1.47	0.98	1.08	1.04
Hydroxyisobutyrate	1.08	1.02	1.03	0.99
Valine	1.03	***0.90***	1.02	0.99
Lipids (LDL)	0.87	1.04	0.96	1.00

Significant differences between groups were determined by two-tailed t tests with Welch’s correction, and are indicated in bold and italicized.

**Table 2:** Concentrations of major endogenous metabolites quantified in rat serum by ^1^H-NMR, and their change in expression in vehicle/IR and SCN^−^-supplemented/IR rats at 4 weeks following surgery.

Metabolite	Chemical Shift (ppm)	Average fold change at 4 weeks: vehicle/sham versus vehicle/IR	Average fold change at 4 weeks: SCN/sham versus SCN/IR	Average fold change at 4 weeks: vehicle/IR versus SCN/IR
Formate	8.52	0.76	1.00	0.84
Cytidine	7.80	0.85	0.93	0.91
Methylhistidine	7.74	0.94	0.86	1.02
Histidine	7.71	0.84	0.82	0.97
Phenylalanine	7.40	0.99	0.96	1.02
Tyrosine	7.17	0.82	0.92	0.90
Lactate	4.11	1.05	1.05	1.07
Isoleucine	3.65	0.96	0.92	1.08
Creatinine/creatinine phosphate	3.03	***0.85***	0.90	0.90
Creatine	3.00	***0.82***	0.88	0.91
Trimethylamine	2.91	***0.88***	0.98	0.92
Methylguanidine	2.81	1.01	0.96	1.05
Methionine	2.64	0.95	0.97	0.97
Glutamine	2.46	0.91	0.95	0.94
Acetoacetate	2.22	1.08	1.16	0.96
Acetyl Groups (1)	2.13	0.92	0.95	0.93
Acetyl Groups (2)	2.06	0.95	0.98	0.95
Acetate	1.91	1.26	0.96	1.09
Leucine	1.72	***0.89***	0.90	0.95
Lipids (VLDL)	1.57	1.08	1.17	0.96
Alanine	1.47	0.94	0.92	0.95
Hydroxyisobutyrate	1.08	1.00	0.93	1.02
Valine	1.03	0.99	0.98	1.00
Lipids (LDL)	0.87	1.02	1.05	0.97

Significant differences between groups were determined by two-tailed t-tests with Welch’s correction, and are indicated in bold and italicized.

**Figure 8:**
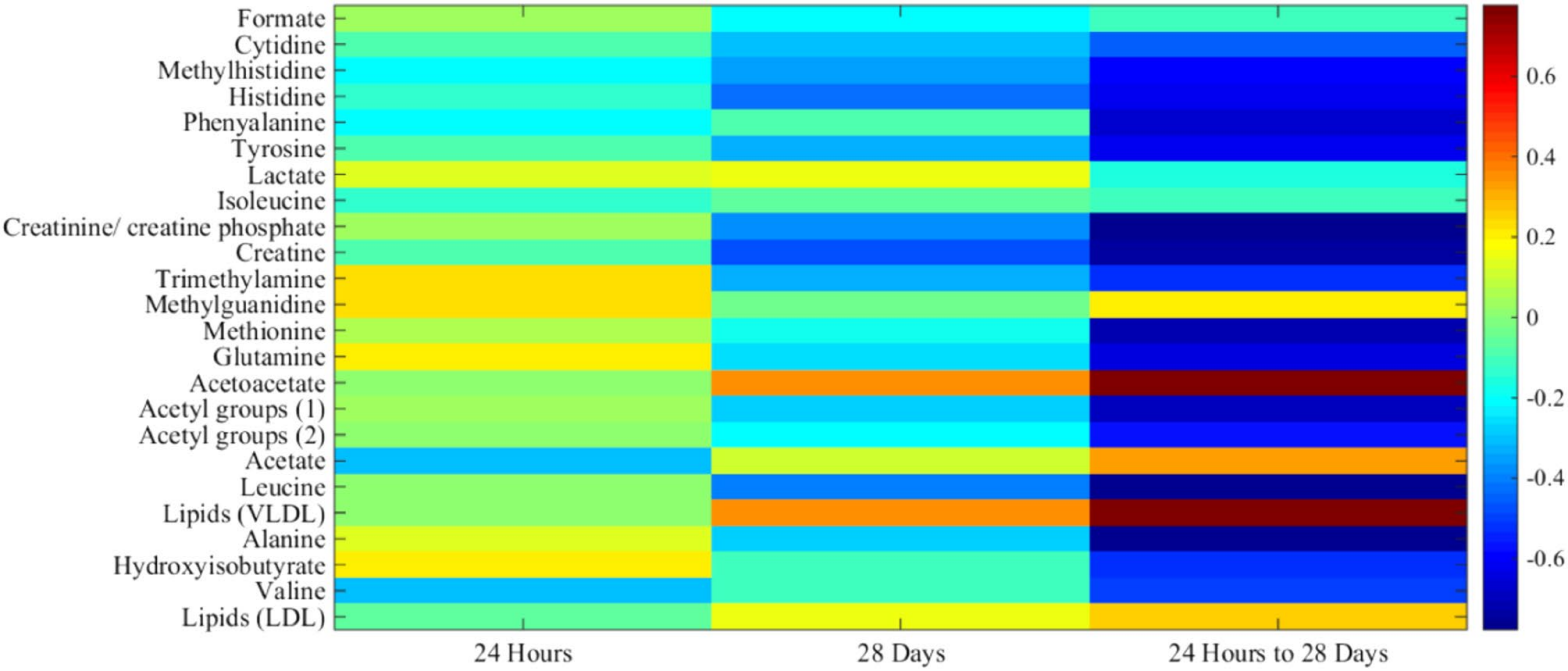
Heat map depicting the correlations (R^2^ values) of major endogenous metabolites measured in rat serum by ^1^H NMR using a Carr-Purcell-Meiboom-Gill (CPMG) spin echo sequence experiments with procedure type. The first column displays correlations for the vehicle/IR animals relative to sham-operated controls at 24 h post-surgery; the second column displays correlations between the vehicle/IR animals and sham-operated controls at 4 weeks (28 days) post-surgery, with hot (e.g. red) colors denoting a positive correlation for the IR cohort. The final column displays correlations between the vehicle/IR animals at 4 weeks (28 days) relative to 24 h post-surgery, with hot colors (e.g. red) denoting a positive correlation.

**Figure 9:**
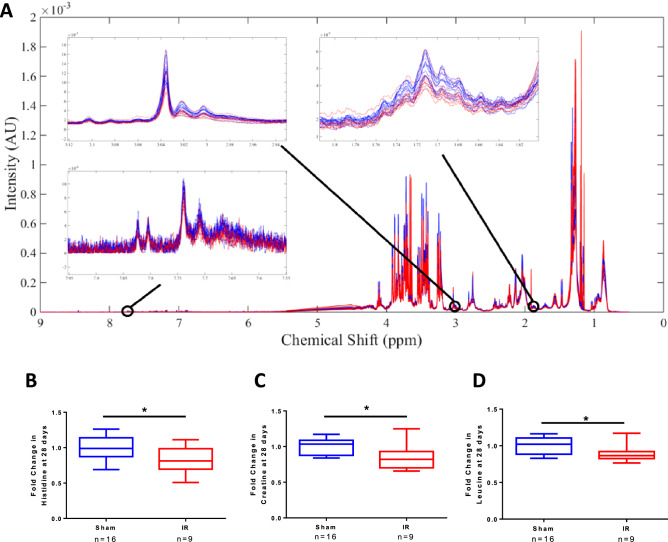
**(A)** ^1^H CPMG NMR spectra coloured by operation type (blue: sham-operated, red: IR) from samples collected from vehicle/sham operated versus vehicle/IR animals at 4 weeks post IR surgery. The insets show the significant reductions in the resonance from histidine, creatine, and leucine (left to right respectively) after IR surgery. **(B)** Fold changes in histidine (left), creatine (middle), and leucine (right) between rats subject to sham-operations or IR injury, at 4 weeks post-surgery. Two-tailed Student’s t-test shows a significant difference in the concentrations of histidine, creatine, and leucine between the sham and IR groups, where * indicates a statistical significance at the p < 0.05 level.

**Figure 10:**
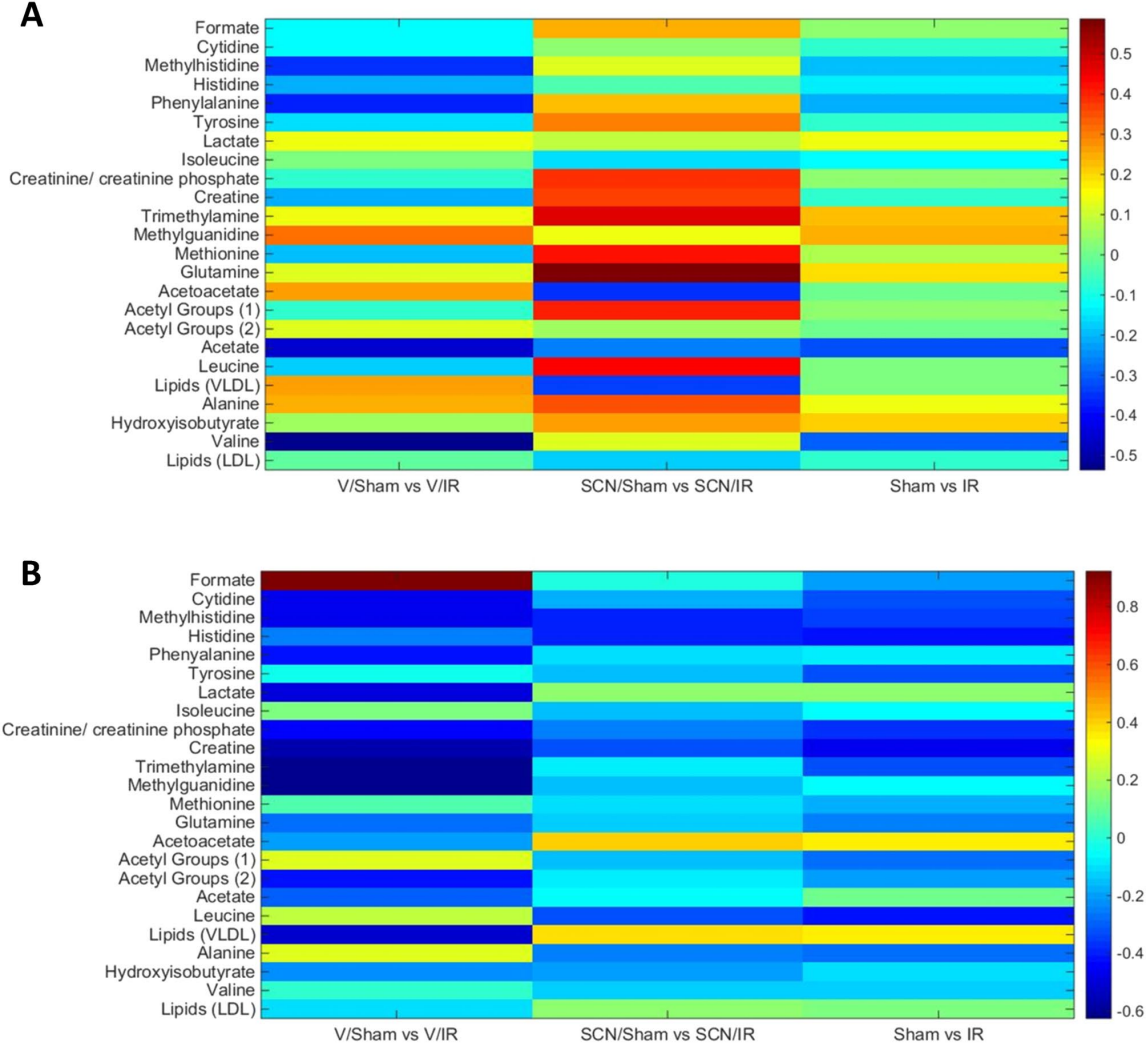
**(A)** Heatmap of major endogenous metabolites quantified in rat serum by ^1^H-NMR, and their change in expression at 24 h post-surgery. The first column shows the fold change in vehicle/IR rats relative to the vehicle/sham controls, the second column shows the fold change in SCN^−^ -supplemented/IR rats relative to the SCN^−^ -supplemented/sham operated controls, and the third column shows the fold change for all IR rats relative to all shams. **(B)** As panel (A) except for animals at 4 weeks post-surgery. In both cases, hot colors (red) indicates an increased fold change, while cold colors (blue) indicates a reduced fold change.

Figures 8, 9 and 10 depict the data for the vehicle/IR group relative to vehicle/sham groups (i.e. the changes arising from IR injury) at 24 h and 4 weeks, and the differences between the 24 h and 4 week groups, in the form of heat maps, where red indicates elevated levels, and blue represents reduced levels, of metabolites relative to respective controls. Statistically-significant fold reductions were seen in the vehicle/IR group relative to vehicle/sham at 4 weeks post-surgery. Creatinine/creatinine phosphate (3.03 ppm) had a fold change of 0.85, creatine (3.00 ppm) had a fold change of 0.82, trimethylamine (2.91 ppm) had a fold change of 0.88, and leucine (1.72 ppm) had a fold change of 0.89 (Fig. 9).

In contrast, the SCN/IR group showed no statistically-significant fold changes relative to the SCN/sham control at 4 weeks post-surgery. No significant differences were detected between the SCN/IR and vehicle/IR groups, nor between the SCN/IR group and the SCN/sham, at this time point. No significant changes in metabolite expression were detected between the vehicle/IR and SCN/IR groups, despite the differences detected between these groups and their respective controls (Figs. 9, 10).

## Discussion

**Figure 11:**
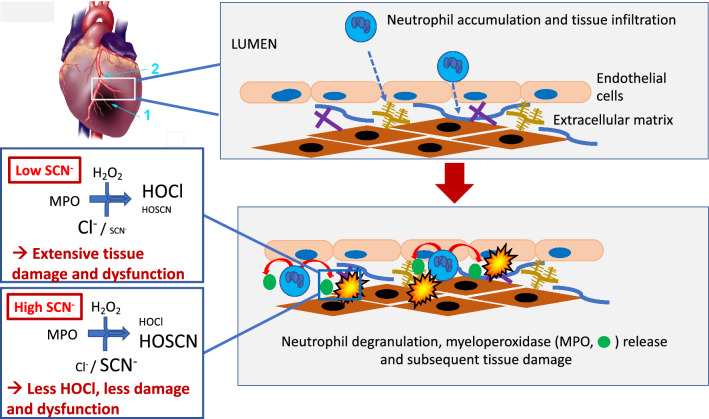
Proposed model of protection by SCN^−^ against IR injury. Occlusion of the left anterior descending artery in rats gives an area of ischemia (1). The site of ligation (2) in indicated. Subsequent release of the ligature results in reperfusion of this area. The restoration of blood flow results in a rapid infiltration of leukocytes, predominantly neutrophils during the initial period, that accumulate in the damaged tissue. Activation of these cells, results in the formation of superoxide radicals (O_2_^−**.**^, via the action of NADPH oxidase-2) and H_2_O_2_ (from O_2_^−**.**^ dismutation), and release of the heme enzyme myeloperoxidase (MPO). MPO utilizes H_2_O_2_ and chloride ions to give the potent oxidant and tissue-damaging species, hypochlorous acid (HOCl). SCN^−^ is a competing substrate for MPO, and elevation of the levels of this anion decreases HOCl formation. Supplementation of SCN^−^ is shown here to limit tissue damage and dysfunction, with this resulting in: reduced infarct size and increased salvage of the area at risk (as determined by MRI imaging); increased left-ventricle end diastolic and systolic areas (echocardiography); decreased modification of tissue proteins and increased collagen content (antibody recognition and histological staining); and decreased metabolic abnormalities (NMR metabolomics). No effects of SCN^−^ on serum markers of inflammation and tissue damage were detected. Heart image adapted, with permission, from Wu et al.^4^.

Acute obstruction of a coronary artery results in acute ischemia in the region of myocardium supplied by that vessel. If the occlusion is released, to prevent permanent ischemic damage, reperfusion occurs resulting in alternative pathways of tissue injury. Both the ischemic and reperfusion phases can potentially result in extensive myocardial damage. Previous studies have provided a wealth of information on the changes that occur as a result of both MI and IR, but less progress has been made in developing therapeutics that modulate these effects at both the tissue and circulating serum metabolome levels (e.g.^11^). The current study examined the hypothesis that elevated levels of SCN^−^, delivered via oral supplementation, would afford protection by diminishing damage induced by HOCl produced by MPO released from infiltrating neutrophils as a result of the ischemic insult. The data obtained indicate that SCN^–^ supplemented rats challenged with IR injury had better outcomes when compared to animals challenged with IR without SCN^−^ supplementation as shown by a reduction in mean infarct area and a greater proportion of salvageable tissue. In addition, the echocardiography data showed that SCN^–^ supplemented rats had less functional damage, as assessed by fractional shortening, and LVED and LVES areas, with these parameters comparable to those determined for the vehicle/sham control rats. In contrast, the IR rats without SCN^–^ supplementation showed a statistically-significant increase in LVED and LVES areas at multiple time points and in multiple heart sections, with these changes being similar to those associated with HF^45,46^. The greater extent of tissue salvage is believed to contribute to the differences in LVED and LVES parameters, with this arising from a protective effect of SCN^−^, throughout the tissue sections evaluated, against oxidant-induced disruption of electrical conductance and tissue integrity, function and remodeling, with the changes being most marked in the mid-section of the heart proximal to the site of ligation (Fig. 11).

As previous studies have reported increased collagen turnover (both synthesis, driven by tumour growth factor-β_1_, and matrix metalloproteinase [MMP]-mediated degradation) in the infarct region following MI/IR injury^53–56^, changes in collagen content were assessed in the IR cohorts 24 h following injury. Previous studies have shown that maintenance of the collagen matrix architecture is essential for the integrity of the infarcted area and myocyte survival^57^, but that excessive collagen deposition and impaired repair of the native extracellular matrix can be deleterious to the tensile strength of the recovered tissue and promotes contractile dysfunction^58^.

Potential associations with SCN^–^ supplementation were examined, as previous research has reported that antioxidant supplementation can reduce collagen synthesis following MI/IR^59^. The level of collagen deposition detected in the vehicle/IR animals were modest, probably as a result of the early time point post-injury that was examined. This is in line with reports that enhanced mRNA expression, and subsequent collagen synthesis and deposition, occur at longer time points after the initial injury (e.g. significant upregulation of Type III procollagen mRNA at day 2, and subsequent collagen deposition with maximal levels at day 14 post infarct^54^). Interestingly, in this study, oral supplementation with SCN^−^ increased the area of positive collagen staining, in contrast to some other interventions^60^.

To examine whether the protective effect of SCN^−^ was associated with decreased HOCl production, the extent of epitope recognition by the mAb 2D10G9, which was raised against HOCl-modified proteins^47^, was assessed. Overall, supplementation with SCN^−^ decreased the extent of 2D10G9 epitope recognition in the hearts following IR injury, consistent with a decreased extent of protein modification by MPO-derived HOCl. Interestingly, this was not the case at the site of ligation, where a similar extent of fluorescence from 2D10G9 epitopes was seen in the absence and presence of SCN^−^, despite the prediction of a high concentration of neutrophils in this region^20^, though this was not assessed which is a shortcoming of this study. This lack of difference may be due to the extensive tissue necrosis at this site (cf. Fig. 3). However, the level of 2D10G9 staining decreased from the site of ligation to the apex, consistent with the greatest extent of neutrophil accumulation at sites proximal to the infarction zone^20^. Examination of the extent of apoptosis and necrosis in the tissues at various time points after IR injury in both the non-supplemented and SCN^– ^dosed animals may shed further light on the mechanisms of damage propagation and its prevention.

The observed suppression of HOCl-induced protein modification is attributed to the competition between SCN^−^ and Cl^−^ for the activated (Compound I) form of MPO, with this favoring SCN^−^ when this is present at elevated levels (~ 110 µM in the serum of the supplemented animals, Fig. 2). Whilst it is possible that this may also arise from direct scavenging of HOCl by SCN^−^ (which occurs with rate constant, *k*, 2.3 × 10^7^ M^−1^ s^−1^ ^61^), the modest concentration of SCN^−^ relative to other biological targets (e.g. mM concentrations of proteins and hence molar concentrations of sidechains) makes this less likely. A limitation of these studies is the lack of data on tissue levels of SCN^−^, though these have been reported to be similar to serum levels^62^.

The negative correlation detected between collagen content and 2D10G9 staining (i.e. HOCl-induced protein modification) is consistent with an increased destruction or turnover of collagen in tissue regions exposed to the highest oxidative insult. Whether this enhanced collagen turnover, or loss, is due to direct oxidative damage by HOCl (as reported in other tissues^63^), via HOCl-mediated activation of latent (pro) MMPs (as described in^64,65^), and / or inactivation of the tissue inhibitors of MMPs (TIMPs)^66,67^, requires further study. A limitation of this study is that collagen levels and potential fibrosis were not quantified at later time points to examine the potential long-term consequences of SCN^−^ supplementation on IR injury. Interestingly, both the non-supplemented IR group and the SCN^–^ supplemented/IR group showed this negative correlation, but the former started at a higher ratio (2D10G9 staining : collagen content) and had a steeper gradient than the SCN^−^ group, consistent with SCN^−^ affording protection, potentially via inhibition of MMP activation by HOCl, or preservation of TIMP activity.

In contrast to the positive effects of SCN^−^ detected in the myocardium, the systemic protective effects of this anion were more limited, with no significant changes detected with SCN^−^ supplementation for ANP, BNP, galectin-3 or C-reactive protein, suggesting that marked protective effects of this anion are limited to locales where there are elevated numbers of activated neutrophils and marked release of MPO such as the infarct region of myocardium. The overall levels of these markers were elevated, as expected, when compared to animals that had not undergone surgery, and in the case of C-reactive protein, the levels diminished significantly between the 24 h and 4 week time points, consistent with the acute nature of this marker and its short (~ 19 h) half-life^52^. Whilst there is likely to be some wash-out of damaged materials into the circulation from the site of damage, any protective effects of SCN^−^ appear to be diluted to a degree such that no marked systemic changes were detected. A limitation of this study is that other markers of inflammation (such as interleukin-6, caspase 3 and NLRP3) were not examined.

Whilst no significant changes were detected in the above markers, limited significant changes were detected in the metabolomic dataset. It is well established that the heart requires a high rate of ATP production to maintain its continuous function, therefore perturbations in ATP-generating processes would be expected to directly affect contractile function^68^, and it is known that there is increased catabolism following acute MI^69^. Available data suggest that myocardial substrate selection remains relatively normal in the early stages of HF, but that in later stages, there is a downregulation in fatty acid oxidation, increased glucose oxidation and glycolysis, reduced respiratory chain activity, and an impaired mitochondrial oxidative flux reserve capacity^70^. Our data are consistent with this hypothesis, as significant reductions in serum histidine, creatine, and leucine levels were detected for the vehicle/IR group at 28 days post-surgery when compared with sham-operated control animals. Previous studies have suggested that the failing myocardium suffers from a significant reduction in total creatine^71,72^, but the mechanism(s) by which alterations in the concentrations of ‘high-energy’ phospho-compounds compromise cardiac energy metabolism and contractility are not well understood^73^. Creatine is not synthesized by cardiomyocytes, but is taken up into the myocardium from serum via a specific Cl^−^- and Na^+^-dependent creatine cotransporter, which has been reported to be down regulated in HF^72^, with this resulting, along with diminished creatine kinase activity, in limited energy reserves in the failing heart^74^. A consequence of decreased phosphoryl transfer via creatine kinase, is an increased cost of mechanical work and decreased contractile reserve, rendering the heart muscle more susceptible to ischemic injury^73,74^. These conclusions are supported by our finding of reduced creatine levels at 28 days post IR surgery, and data from otherwise normal rat hearts where creatine kinase activity or creatine levels were decreased^74^.

Impairment of mitochondrial electron transport resulting from IR injury can result in compensatory changes^75^, including catabolism of proteins, polysaccharides and fats, to amino acid, monosaccharide, and fatty acid constituents, with subsequent oxidation of these species to produce acetyl CoA. Whilst amino acids are only a minor fuel under normal circumstances, these reactions appear to become more important during HF^76,77^. Histidine and leucine participate in oxidative metabolism via two different pathways. As a glucogenic amino acid, histidine can be converted to glucose through gluconeogenesis, with the glucose then used to generate ATP and pyruvate via glycolysis^77^. Subsequent pyruvate decarboxylation results in acetyl CoA formation for the tricarboxylic acid cycle. In contrast, as a branched ketogenic amino acid, leucine oxidation gives rise to thiolytic cleavage of β-ketoacyl or β-hydroxy acylCoA derivatives to generate acetyl CoA^77,78^. Thus, the reductions in histidine and leucine detected in the current study are proposed to arise via an upregulation of these catabolic pathways in the wake of IR injury, as a compensatory mechanism for impaired mitochondrial ATP production. Such defects are increasingly considered an important determinant in the progression of HF following IR injury^73,79^. In each of these cases, the deficits induced by the IR injury when compared with the control (sham operated) animals were not detected when the non-supplemented IR injury group were compared to the SCN^–^ supplemented group, consistent with less marked changes when the animals were supplemented with SCN^−^.

There is increasing interest in the use of SCN^−^ as a means to decrease the extent of HOCl-induced tissue injury in chronic inflammatory pathologies, including cystic fibrosis and atherosclerosis (reviewed^23,80,81^). Supplementation with SCN^−^ favors the production of HOSCN over HOCl, with the former being (generally) less toxic to mammalian cells than the latter (e.g.^37,43,44,82,83^). In particular, and of relevance to the current study, reagent HOSCN has been shown to have less potent effects than HOCl on the H9c2 cardiac myoblast cell line, with lower levels of mitochondrial dysfunction, Ca^2+^ overload and apoptotic and necrotic cell death, and evidence for HOSCN-mediated induction of pro-survival signaling cascades that result in a restoration of myoblast function^44^. Overall, the data reported here suggest that SCN^−^ supplementation may decrease IR injury following MI, and help rationalize the reported association between high SCN^−^ levels and decreased all-cause mortality in patients who survived an initial MI^39^. A proposed model for the observed protective effects of SCN^−^ is presented in Fig. 11. This effect of SCN^−^ is of potential therapeutic significance, given the ease with which serum and tissue levels of SCN^−^ can be enhanced via dietary modifications^84,85^.

## Materials and methods

### Rat model of myocardial infarction

Male Sprague–Dawley rats (weight 250 to 300 g) were acclimatized for a period of 1 week prior to the start of the experimental protocol. After the acclimatization period, the rats were divided in a random manner into vehicle controls (2 groups, n = 16 each) and 10 mM SCN^–^ treated groups (2 groups, n = 16 each), with the SCN^−^ (as NaSCN) dissolved in drinking water, for 1 week of preconditioning prior to surgery. This dosing period has been previously shown to result in significant and sustained elevations in plasma SCN^−^ concentrations^25,26^. Both food and water were available ad libitum. All procedures were approved by the Royal North Shore Hospital Animal Care and Ethics Committee (approval no. 5405-005A) and the study was carried out in compliance with the ARRIVE guidelines. Myocardial IR was induced as described previously, using an open‐chest method^6^. Ischemia was induced for 30 min by transient suture ligation of the left anterior coronary artery approximately 2 to 3 mm distal to the junction of pulmonary artery and left atrial appendage. Sham operated animals underwent the same surgical procedure and ligation placement, but the ligature was not tightened in these animals. For the IR animals, the ligature was released after the 30 min period. For each group, the rats were then allowed to recover for either 24 h or 4 weeks before sacrifice. Immediately prior to sacrifice, rats destined for MRI analysis (n = 8 per group with 24 h recovery period) had the LAD ligature retightened. The MRI contrast agent gadobenate dimeglumine (Multihance, 0.5 M, 0.4 mL kg^−1^) was infused via a tail vein cannula 10 min before sacrifice, and 2 min before sacrifice, iron microparticles (Dynabeads MyOne tosylactivated; 4.5 mg kg^−1^) were also infused via the tail vein cannula as described previously^86^. These agents modulate the T1 (gadolinium) or T2/T2* (iron oxide) relaxation of adjacent water protons. The remaining animals that were not infused with contrast agents had blood samples collected for analysis, including SCN^−^ concentrations, biomarkers of HF, as well as metabolomic analyses. The overall experimental protocol is indicated in Fig. 1. All methods were performed in accordance with the relevant guidelines and regulations.

### Blood collection

In animals that were not infused with the contrast agents, ~ 4 mL of blood was collected, at the time of sacrifice from the inferior vena cava into tubes and allowed to clot. These were then centrifuged for 10 min, and the resulting serum stored at -80 °C for analysis. The hearts were also collected and immediately fixed in 10% neutral buffered formalin (NBF) for 24 h, after which they were embedded in 2% agar prior to MRI imaging and subsequent histological analysis.

### Magnetic resonance imaging

All samples were scanned on a 400 Hz (9.4 T) Bruker Scanner in a similar manner to that reported previously^86^. Samples were placed in the center of the coil, and scanner gain was set and tuned to each sample individually. A pilot scan was used as a reference to align both sequences. A T2Star sequence (TR/TE = 40/15 ms, FOV = 15/15/18 mm, matrix size = 128/128/64, Spatial Resolution = 0.117/0.117/0.28 mm, scan duration 16 min) was used to show the location of the iron particles^86^. A T1 inversion recovery sequence (TR/TE/TI 1000/12.5/200 ms; FOV 15/15/18 mm; matrix size 96/96/32; spatial resolution 0.15/0.15/0.56 mm; scan duration 51 min) was used to confirm the location of the gadolinium. Data analysis was performed in MATLAB with in-house software. Data from the scanner was reconstructed into .mat format from the raw data fid files for every sequence. All sequences were read into MATLAB and interpolated in the in-plane direction to a finer grid (256/256). T2star sequences were used to calculate left-ventricle volume. Both epicardium and endocardium borders were manually delineated from each slice and used to create a mask. The number of voxels within the mask was then multiplied by the voxel volume to calculate the left-ventricle volume. All T2 star images were subjected to a threshold from a minimum to 0.66 of maximum pixel intensity to aid segmentation. T2star sequences were then used to define the total reperfusion volume from the delineation of the border of the iron microparticles. The total reperfusion mask was clipped by the left-ventricle mask to ensure that only the ventricular area was analyzed. The total reperfusion volume was calculated in a similar manner.

The infarct volume was calculated from the real reconstruction of the T1 inversion recovery sequence. Pixel intensity was squared to aid with segmentation and account for variation in gadolinium intensity from sample to sample. All T1 IR images were subject to a threshold from minimum to 0.10 of maximum pixel intensity, to aid segmentation. The area of gadolinium was defined and mask was then created, again this mask was clipped by the left-ventricle mask to ensure the area remained within the left ventricle. The volume was then calculated as described above. The salvageable volume was calculated by subtracting the infarct volume from the total reperfusion volume. All 3 parameters (total reperfusion, infarct and salvage) were also normalized by left-ventricle volume; salvage volume was also normalized to the total reperfusion volume. Results were output to an excel spreadsheet for further analysis and the observer was blinded to the groups until the statistical analysis stage.

### Echocardiography

2D and M-mode images of a short-axis view at the mid-papillary level were recorded prior to surgery, and 48 h, 1, 2 and 4 weeks after surgery, using a 6.5 MHz transducer. Left ventricular diameters in diastole and systole were measured from M-mode recordings. Fractional shortening was calculated as (LV diastole-LV systole)/(LV diastole) × 100.

### Histology

For tissue sectioning, rat hearts were immersed in OCT compound (Tissue-Tek, Sakura) and frozen at -20 °C. Frozen samples were serially sectioned from the apex to the coronary artery ligation, starting with a 500 µm thickness from the apex, followed by 10 consecutive transverse sections of 10 μm thickness. This pattern was repeated at 500 µm intervals up to the site of ligation, resulting in a series of sections (60–90, depending on the heart size and precise site of ligation) at different distances to the ligation (defined as 0, 600, 1200, 1800, 2400, 3000, 3600, 4200, 4800 µm to the ligation). One section from each group of 10, was fixed and stained using a Trichrome Stain Kit (Abcam), followed by dehydration in ethanol, clearance in xylene and mounting in resin (Pertex, VWR). Brightfield images were obtained using a Zeiss Axio Scan.Z1 microscope with a 10x/0.45 object, with images captured using a CCD HV-F202CLS camera (Hitachi) at 4.4 µm/pixel. Multi-image stitching was performed using a 10% tile overlap.

Adjacent slices were used to quantify HOCl-induced damage. Sections were fixed, washed, blocked and incubated with a murine primary monoclonal antibody 2D10G9 (1:200 dilution), then an Alexa Fluo 594 secondary antibody (1:1000 dilution). DAPI was used to detect nuclei. Sections were then mounted in ProLong Gold Antifade Mountant (P36930, Thermo Fisher) and allowed to dry overnight in the dark. The stained slides were examined under a fluorescent microscope (Zeiss Axio Scan.Z1) with a Plan Apo 20 × 0.8 NA objective. A Semrock F66-887 multiband filter cube was used to image two channels, with the blue channel used for DAPI, and the red channel for 2D10G9. A Zeiss AxioCam MRm CCD with a 6.45 µm per pixel camera was used for image capture. Multi-image stitching was performed using a 10% tile overlap.

Image analysis was performed using ZEN Blue 3.2 software. For collagen staining, the Intellesis machine learning module included in the software package was used. This is a Random Forrest pixel classifier that extracts 33–256 pixel features. Iterative rounds of training of the machine learning tool was done by annotation of the ground truth, achieving a successful segmentation of red-colored myocardium, blue/purple-colored collagen and the background. The same methods and settings were applied to all sections. For 2D10G9 staining, the regions of interest were obtained by segmentation by intensity thresholding, setting common intensity thresholds at 150 and 350 grayscale levels for the background and the tissue, respectively, for the whole dataset. The area of stained tissue and the mean intensity were determined.

### Serum analyses

SCN^−^ concentrations were quantified by ion-exchange chromatography^26,87^. Serum samples were mixed 1:1 (v/v) with acetonitrile, followed by centrifugation (5000*g*, 4 °C, 7 min) to remove precipitated proteins. The supernatant was subsequently filtered through 0.2 μm centrifuge filters (5000*g*, 4 °C, 3 min; Pall Nanosep MF, 500 μL capacity). Samples (25 μL) were then injected on to an IonPac AS16 column (4 × 250 mm) via an AG16 guard column (4 × 50 mm) fitted into a Dionex chromatography system consisting of an AS3500 autosampler, GP40 gradient pump, ASRS 300 anion suppressor (4 mm) in AutoSuppression recycle mode, and an ED40 electrochemical detector with DS3 detector stabilizer. Ions were eluted over 12 min with 50 mM potassium hydroxide in water with a flow rate of 1.5 mL min^−1^ and a suppressor current of 186 mA. Peak areas were quantified using Chromeleon Chromatography Studio software (version 7), against a standard curve generated using commercial NaSCN (0–200 μM).

ANP, BNP, CRP, and galectin-3 were quantified using commercial kits (Atrial Natriuretic Peptide EIA Kit, Brain Natriuretic Peptide EIA Kit, and rat C-Reactive Protein ELISA Kit, all from Sigma-Aldrich; and rat Galectin-3 ELISA from Ray Biotech) in accordance with the manufacturer’s instructions.

### Sample preparation for metabolic profiling

Sample preparation and acquisition methods were based on published protocols^88^. Serum aliquots (300 µL) were mixed with 300 µL of aqueous (20% D_2_O) phosphate buffer solution (0.075 M NaH_2_PO_4_, pH 7.4) also containing 0.1% (w/v) sodium azide and 1 mM 3-trimethylsilyl-1-[2,2,3,3-^2^H_4_] propionate (TSP). The samples were then centrifuged at 3000*g* for 10 min, and 500 µL aliquots of the supernatants transferred into 5 mm NMR tubes for ^1^H NMR analysis.

### Acquisition of ^1^H NMR spectra

^1^H NMR spectra were acquired using a Bruker Avance III 400 MHz spectrometer equipped with a 5 mm broad-band inverse configuration probe, operating at 400.13 MHz, for ^1^H spectroscopy at 310 K. Samples were analysed in a random manner using an automatic 24 sample SampleCase changer system. Serum samples were analyzed using two experiments run sequentially, involving an initial water-suppressed 1D NMR NOESYPRESAT pulse sequence (256 transients), followed by a Carr-Purcell-Meiboom-Gill (CPMG) spin echo sequence with water pre-saturation (196 transients)^88^. Both experiments included irradiation of the solvent (water) resonance that was applied using a pre-saturation delay (2 s). The pulse sequence parameters including the 90° pulse (12 µs), pulse frequency offset (1,890.6 Hz), receiver gain (90.5), and pulse powers were optimized on a representative sample and then kept fixed for the entire cohort. All spectra were acquired with a spectral width of 20 ppm. The data were processed with an exponential line broadening of 0.3 Hz prior to Fourier transformation, which were collected with approximately 32 k real data points.

### NMR spectral data processing

Data [-1.0 to 10.0 ppm] were imported into MATLAB R2014b software (MathWorks, Natick, MA), after this they were automatically phased, baseline corrected and referenced to the TSP peak (0.00 ppm) using Topspin 3.2. To reduce analytical variation between samples the residual water signal (4.67–4.98 ppm) was truncated from the data set. Normalization of each spectrum was performed across the sample cohort by use of probabilistic normalization. Assignment of endogenous serum metabolites was made by reference to literature data^89–92^. Following the pre-processing of the NMR data, multivariate statistical analysis was performed using MATLAB 2014b. Principal component analysis (PCA)^93^, was used to facilitate spectral assignments and trends in the metabolite profiles. MATLAB was also used to generate orthogonal partial-least squares discriminant analysis (OPLS-DA) models and to statistically recouple variables (SRV) to quantify metabolites for univariate analysis^94^. All the OPLS models were run through random permutation testing to assess the validity of the supervised model.

### Statistical analyses

Data are expressed as mean ± standard error of the mean (SEM) from at least 6 animals per group: exact numbers are presented in the individual figures and tables. Statistical analyses were undertaken using Prism (versions 6 and 8; GraphPad software, San Diego, CA, USA). For pair comparisons, unpaired t tests with Welch’s correction were employed as indicated in the figure legends. For multiple comparisons, one-way and two-way analysis of variance (ANOVA) with post hoc tests, as indicated in the figure legends, were used to assess differences. Differences were considered to be statistically significant at the p < 0.05 level.

## Supplementary Information

Supplementary Information.

## Abbreviations

AAR: Area at risk
ANP: Atrial natriuretic peptide
BNP: Brain natriuretic peptide
CRP: C-reactive protein
HOCl: The physiological mixture of hypochlorous acid and its anion ^−^OCl
HF: Heart failure
HOSCN: The physiological mixture of hypothiocyanous acid and its anion ^–^OSCN
IR: Ischemia–reperfusion
LAD: Left anterior descending
LVED: Left-ventricle end diastole
LVES: Left-ventricle end systole
MPO: Myeloperoxidase
MI: Myocardial infarction
NBF: Neutral buffered formalin
NMR: Nuclear magnetic resonance
SCN^−^: Thiocyanate
