# Supplementary material for: Oral pre-treatment with thiocyanate (SCN−) protects against myocardial ischaemia–reperfusion injury in rats
Source: Sci Rep. 2021 Jun 16;11:12712. doi: 10.1038/s41598-021-92142-x (PMC8209016; doi:10.1038/s41598-021-92142-x)
Supplement: Supplementary file 1 — Supplementary Information. [file 41598_2021_92142_MOESM1_ESM.pdf]

Oral pre-treatment with thiocyanate (SCN^-) protects against myocardial ischaemia-reperfusion injury in rats

Luke Hall[1,2,3], Chaouri Guo[3], Sarah Tandy[2,4], Kathryn Broadhouse[1,2], Anthony C Dona[2,4], Ernst Malle[5], Emil D. Bartels[6], Christina Christoffersen[3,6], Stuart M. Grieve[2], Gemma Figtree[2,4], Clare L. Hawkins[3] and Michael J. Davies[1,2,3] *

[1]The Heart Research Institute, Newtown, NSW 2042, Australia

[2] School of Medicine, University of Sydney, Sydney, NSW 2006, Australia

[3] Department of Biomedical Sciences, Panum Institute, University of Copenhagen, Blegdamsvej 3, Copenhagen 2200, Denmark

[4] Kolling Institute of Medical Research, Northern Medical School, University of Sydney, NSW 2065, Australia

[5] Division of Molecular Biology and Biochemistry, Gottfried Schatz Research Center, Medical University of Graz, Graz, Austria

[6] Department of Clinical Biochemistry, Copenhagen University Hospital Rigshospitalet, Copenhagen, Denmark

* Corresponding author

Email address: davies@sund.ku.dk

Supplementary Data

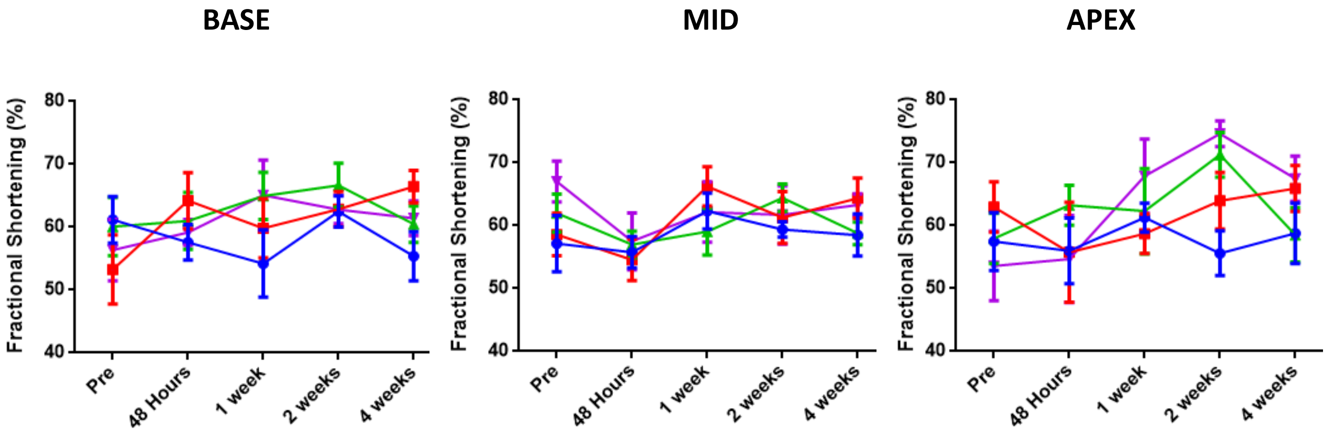

Supplementary Figure 1 Fractional shortening in the base (left panel), mid (middle panel) and apex (right panel) sections from pre-surgery to 4 weeks post-surgery. The red line and symbols are data from the vehicle/sham group; the blue line and symbols are data from the vehicle/IR group; the pink line and symbols are data from the SCN^--treated/sham group; and the green line and symbols are data from the SCN^--treated/IR group. No significant differences were observed between any of the groups at any time point using a two-way ANOVA and Dunnett's Multiple Comparison Test.

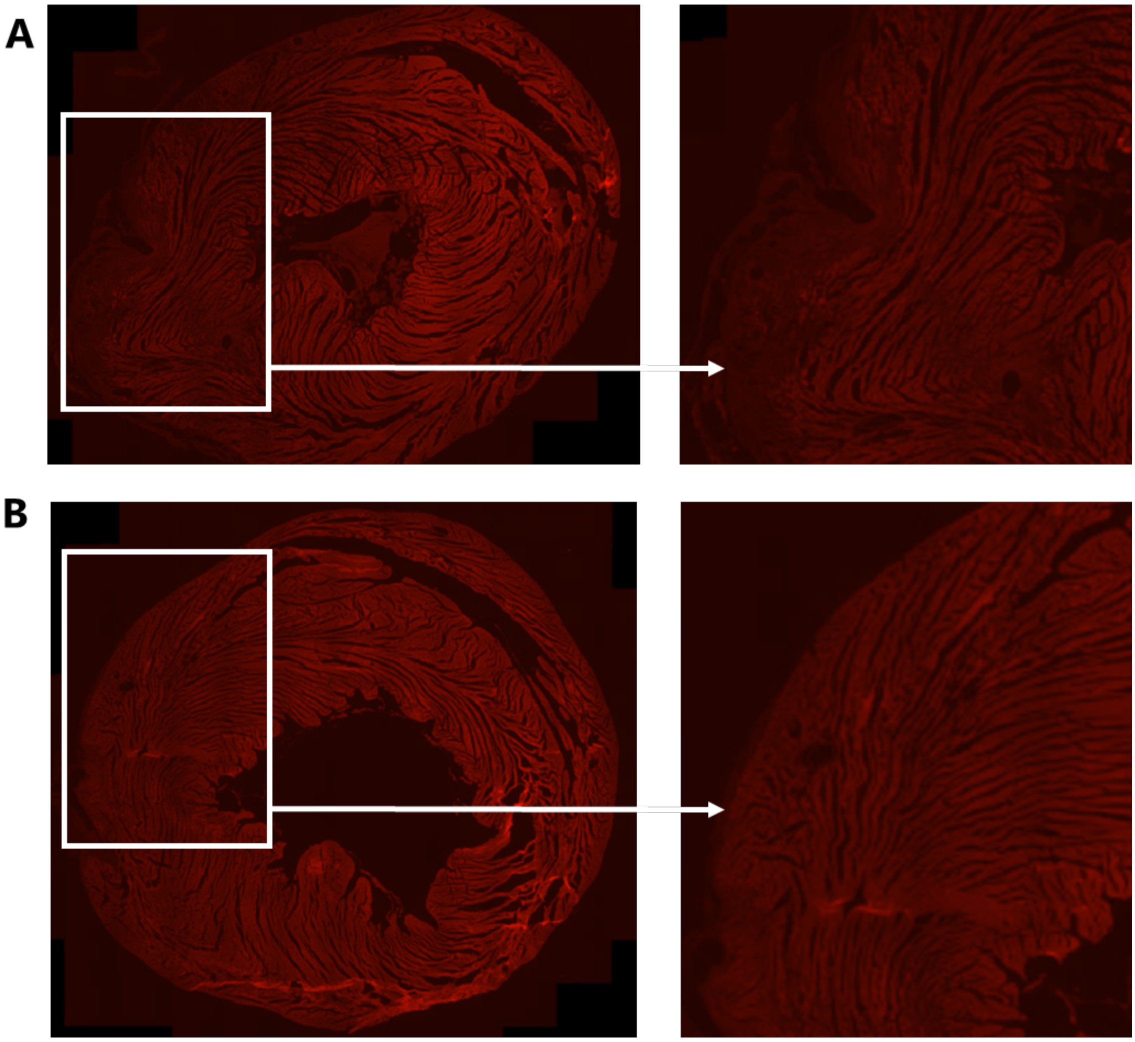

Supplementary Figure 2. SCN$^-$-supplementation does not change the extent of 2D10G9 recognition at the site of ligation. Figure shows representative images taken from sections at the site of ligation from: (A) a vehicle/IR rat and (B) a SCN$^-$-treated IR rat, showing fluorescence from 2D10G9 antibody recognition of HOCl-modified proteins. The expanded images (right panels) show the site of ligation as determined from the histology images in Figure 5.

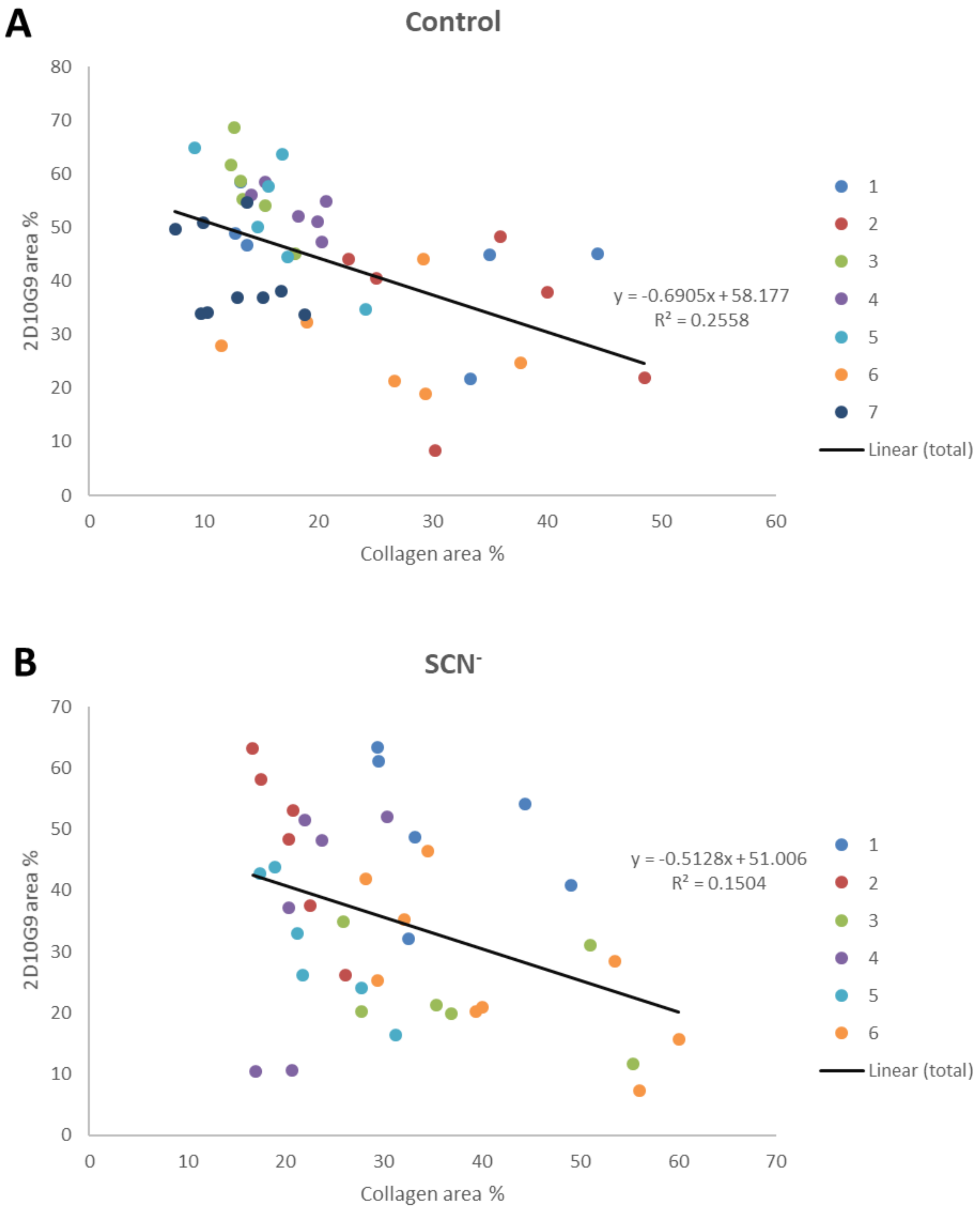

Supplementary Figure 3. Correlation of the collagen content and extent of 2D10G9 epitope recognition. The graphs show a linear regression analysis of the percentage fluorescence from recognition with the monoclonal antibody 2D10G9 (which recognizes HOCl-modified proteins) relative to total tissue area shown by the background fluorescence in the section versus the percentage collagen area of the total stained tissue in the section, for panel (**A**) the vehicle/IR rats (n = 7 animals) and (**B**) the SCN^--treated IR rats (n = 6 animals). The black lines show a regression analysis with the regression formula shown in the figure. Different colours represent multiple tissue sections taken between the ligation site and apex of the heart in individual rats.

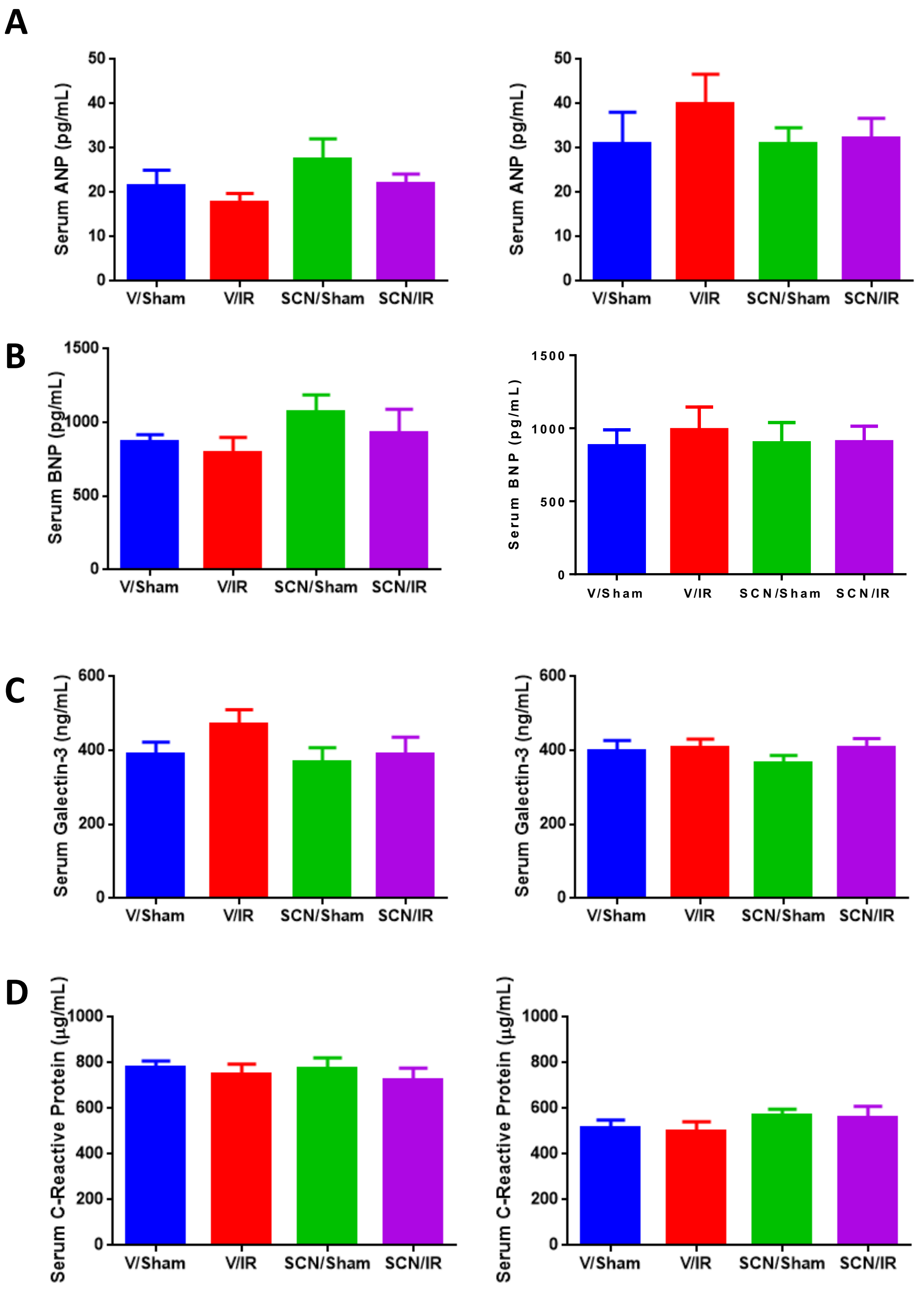

Supplementary Figure 4. Levels of serum markers of tissue damage and inflammation at 24 h (left column) and 4 weeks (right column). **(A)** Serum ANP. **(B)** Serum BNP. **(C)** Serum galectin-3. **(D)** Serum C-reactive protein. In each case no significant differences were observed using a one-way ANOVA and Newman-Keul's Multiple Comparison Test.

Supplementary Table 1. Correlation data for the percentage of collagen area compared to total tissue area against percentage of 2D10G9 (HOCl-modified protein) staining of tissue compared to total tissue area. Data are presented for the individual animals and also for the total group. Negative numbers indicate a negative correlation, positive numbers a positive correlation. * Indicates a statistically-significant value at the $p < 0.05$ level. r values are the determined correlation coefficients.

	Vehicle/IR animals			SCN^- -treated/IR animals		
Rat no.	slope	p	r	slope	p	r
1	-0.4332	0.3181	0.4951	-0.5214	0.4832	0.3601
2	-0.4257	0.6038	0.2707	-3.9274	0.0009 *	0.9757
3	-3.3144	0.0176 *	0.8895	-0.2734	0.4453	0.3895
4	-0.4835	0.1186	0.7039	3.0946	0.1129	0.7114
5	-1.9729	0.0504	0.8106	-1.8808	0.0081 *	0.9255
6	-0.1251	0.8144	0.1244	-0.7215	0.0379 *	0.6945
7	-0.7835	0.3569	0.3493			
total	-0.6905	0.0004 *	0.5057	-0.2933	0.0147 *	0.3878
